# NeuroQ: Quantum-Inspired Brain Emulation

**DOI:** 10.3390/biomimetics10080516

**Published:** 2025-08-07

**Authors:** Jordi Vallverdú, Gemma Rius

**Affiliations:** 1 Philosophy Department, ICREA-UAB, Bellaterra, 08193 Barcelona, Spain; 2 Institut de Microelectrònica de Barcelona, IMB-CNM (CSIC), Bellaterra, 08193 Barcelona, Spain; gemma.rius@csic.es

**Keywords:** quantum-inspired brain emulation, stochastic mechanics, FitzHugh–Nagumo model, neuronal planck constant, hamiltonian simulation

## Abstract

Traditional brain emulation approaches often rely on classical computational models that inadequately capture the stochastic, nonlinear, and potentially coherent features of biological neural systems. In this position paper, we introduce NeuroQ a quantum-inspired framework grounded in stochastic mechanics, particularly Nelson’s formulation. By reformulating the FitzHugh–Nagumo neuron model with structured noise, we derive a Schrödinger-like equation that encodes membrane dynamics in a quantum-like formalism. This formulation enables the use of quantum simulation strategies—including Hamiltonian encoding, variational eigensolvers, and continuous-variable models—for neural emulation. We outline a conceptual roadmap for implementing NeuroQ on near-term quantum platforms and discuss its broader implications for neuromorphic quantum hardware, artificial consciousness, and time-symmetric cognitive architectures. Rather than demonstrating a working prototype, this work aims to establish a coherent theoretical foundation for future research in quantum brain emulation.

## 1. Introduction

The human brain exhibits extraordinary computational power and flexibility, characterized by robustness, energy efficiency, and adaptability. Classical brain emulation efforts—often realized through artificial neural networks or neuromorphic chips—rely on deterministic approximations of neural activity [1]. While useful, these models typically fail to capture the intrinsic stochasticity, coherence, and potential retrocausal features observed in real neural systems [2,3,4]—a perspective increasingly supported by quantum cognitive models and stochastic interpretations of biological systems [5,6].

The intersection of quantum computing and neuroscience has recently gained momentum under what is loosely called “neuroquantum” research. These efforts aim to either use quantum models to simulate brain-like functions or explore whether certain brain processes exhibit quantum characteristics [7,8,9].

Notable examples include:

- **Quantum neural network architectures** that model neural computation via quantum spiking networks or quantum LSTM modules, often applied to classification or memory tasks [10,11].
- **Quantum deep learning in neuroinformatics**, where quantum circuits are used to process neuroimaging data [12].
- **Quantum-classical hybrid models** of brain activity using spin systems and classical fields to simulate multiscale dynamics [8].
- **Quantum neuromorphic hardware proposals**, including oscillator networks and variational circuits for mimicking synaptic behavior [13,14].

These studies demonstrate creative integrations of quantum theory with artificial neural systems. However, they often remain heuristic or task-oriented, with limited grounding in biological models or biophysics.

In contrast, NeuroQ proposes a foundationally different approach:

1. **Biophysical grounding:** Our model derives from the FitzHugh–Nagumo (FHN) neuron equations and incorporates noise following Nelson’s stochastic mechanics [2,3]. This leads directly to a Schrödinger-like formulation of neural evolution, offering formal, physical justification for the quantum analog.
2. **Quantum simulation based on dynamics:** Rather than abstracting neurons as logic units, NeuroQ encodes the stochastic Lagrangian of neural states into Hamiltonians for quantum simulation via Trotterization or variational quantum eigensolvers (VQE).
3. **Conceptual depth:** The framework enables exploration of retrocausal inference, time-symmetry in cognition, and probabilistic agency—issues relevant to philosophy of mind and consciousness studies [15,16].
4. **Testability:** NeuroQ allows empirical validation through estimation of a “neuronal Planck constant” ($\hat{ћ}$) from membrane potential variance, and through neural dynamics observable via patch-clamp or MEA recordings.

Whereas other models often repurpose quantum methods to enhance AI, NeuroQ uses quantum formalisms to better understand the brain itself. This makes it not only an engineering proposal but also a contribution to theoretical neuroscience and cognitive science. Recent advances in stochastic mechanics and quantum-inspired models have prompted renewed interest in whether some aspects of brain function can be interpreted within a quantum-like framework. This view aligns with the growing recognition of quantum-like structures in cognition and biology [6]. Building on Nelson’s seminal work [3], which derives a Schrödinger-like equation from Newtonian mechanics infused with Brownian motion, and recent reformulations of neuron models such as FitzHugh-Nagumo in stochastic quantum terms [2], a new path for brain emulation emerges.

NeuroQ aims to formalize and simulate neural dynamics within such a quantum-inspired paradigm. By mapping noisy membrane potential fluctuations to wave functions and simulating evolution via quantum circuits, we propose a biologically grounded and computationally novel model for brain emulation.

Quantum computing offers several potential advantages for brain simulation:

- **Exponential state-space representation:** Qubits can represent superpositions of neural states, enabling modeling of massively parallel probabilistic processes.
- **Natural noise modeling:** Quantum noise may more faithfully mirror the inherent randomness in ion channel activity or synaptic transmission.
- **Causality reversal and time-symmetric inference:** Time-reversible quantum dynamics allow for retrocausal modeling, a possibility in complex feedback systems in the brain [15,17].
- **Energy efficiency:** Quantum processes, especially in analog or adiabatic systems, may surpass classical neuromorphic chips in efficiency for certain tasks [1].

Unlike traditional AI models that treat neurons as deterministic switches, NeuroQ embraces both the stochastic and potentially coherent nature of real neural substrates. Recent advances in quantum machine learning (QML) have shown that hybrid models combining parameterized quantum circuits with neural networks can improve performance on noisy or structured data [18,19]. NeuroQ complements this line of research by grounding quantum circuit design in biologically inspired stochastic neuron dynamics, offering a principled alternative to purely heuristic QML strategies. Our approach complements recent efforts to integrate machine learning and neuroscience [7], while exploring the fundamental physics underlying cognition.

This paper introduces the mathematical formalism, simulation strategies, and architectural considerations for implementing NeuroQ on quantum computing platforms. Rather than presenting an empirical implementation, this work is intended as a position paper—a conceptual roadmap laying the foundation for future quantum neural emulation research.

**Clarification on Terminology.** Throughout this paper, we use terms such as “quantum-like”, “quantum-inspired”, and “quantum simulation” to describe our modeling framework. These do not imply that the brain itself operates via genuine quantum entanglement, superposition, or tunneling at the molecular level. Rather, the NeuroQ model derives a Schrödinger-type equation from classical stochastic neuron dynamics via Nelson’s theory, producing a *formal analogy* with quantum systems. The use of quantum computing tools (e.g., Hamiltonian simulation, variational circuits) serves as a computational platform for emulating these dynamics, not as a literal assertion of physical quantum processing in the brain. This distinction aligns with prior clarifications in the literature on quantum-like cognition [20] and helps situate our model within a grounded epistemological framework.
**Relation to Quantum Cognition.** NeuroQ also differs from quantum cognition models that apply quantum probability theory to explain decision-making, perception, and ambiguity in human behavior [21,22]. These approaches are largely phenomenological, focusing on cognitive paradoxes (e.g., order effects, violation of classical logic) without positing a biophysical mechanism. In contrast, NeuroQ begins from a physiological model of the neuron (FitzHugh–Nagumo) and maps its stochastic evolution to a Schrödinger-like formalism using Nelson’s mechanics. This grounds probabilistic inference not in abstract logic, but in a structured physical process potentially amenable to experimental validation.

## 2. Background and Related Work

### 2.1. FitzHugh-Nagumo (FHN) Model

The FitzHugh-Nagumo (FHN) model is a reduced dynamical system originally formulated to capture the essential features of neuronal spiking, derived from the more complex Hodgkin-Huxley equations. It simplifies neuronal dynamics into a two-variable system: a fast variable representing the membrane potential and a slow recovery variable accounting for ion channel kinetics [23,24]. Despite its simplicity, the FHN model exhibits nonlinear excitability, threshold behavior, and limit cycles—properties crucial for modeling spiking activity and subthreshold oscillations in neurons. Its mathematical tractability makes it an excellent foundation for introducing stochastic perturbations and exploring quantum analogs of biological computation. While the FitzHugh-Nagumo model abstracts away many biophysical details, it serves as a tractable foundation for incorporating more realistic mechanisms. Future refinements of the NeuroQ framework could integrate specific ion channel models (e.g., Hodgkin–Huxley-type conductances), synaptic current dynamics, or neuromodulatory influences through time-varying input currents or parametric modulation of $\hat{ћ}$. These additions would enable richer modeling of spike initiation, adaptation, and plasticity, bringing the framework closer to biological neuronal behavior without sacrificing its quantum-inspired structure.

### 2.2. Stochastic Mechanics and Nelson’s Theory

In classical physics, stochastic processes are typically treated as sources of noise or randomness external to deterministic laws. However, Edward Nelson’s stochastic mechanics recasts Brownian motion as a fundamental ontological feature that gives rise to quantum behavior [3]. By modeling particles undergoing Brownian motion with time-symmetric diffusion, Nelson derived a form of the Schrödinger equation without invoking wavefunction collapse or hidden variables. In the context of neural modeling, this approach allows one to reinterpret intrinsic noise in neurons—not as a limitation, but as the substrate for emergent quantum-like dynamics.

Recent work by Ghose and Pinotsis [2] applies this theory to the FHN model, deriving a quantum potential and effective Schrödinger-like dynamics for membrane voltage trajectories. This opens a new pathway for understanding how stochastic coherence in neurons might resemble quantum phenomena such as tunneling, interference, or entanglement analogs in population-level neural coding.

### 2.3. Quantum Neural Models and Existing Approaches

Quantum neural models span a diverse spectrum, from quantum-inspired learning algorithms to fully quantum circuit-based architectures. Quantum neural networks (QNNs) leverage superposition and entanglement to represent information more compactly and perform certain computations more efficiently than classical networks [10]. Models such as quantum spiking networks or quantum Boltzmann machines have been proposed, yet most neglect biologically relevant noise and biophysical constraints.

On the hardware side, recent work by Tacchino et al. [13] demonstrated the implementation of artificial neurons using actual quantum processors, marking a step toward neuromorphic quantum computing. However, these models remain largely symbolic or numerical, without integrating physical models of neural dynamics.

By embedding the FHN model in a Nelsonian framework, our approach introduces a biologically grounded route to quantum brain emulation. This unites stochastic neurodynamics, quantum simulation, and cognitive modeling into a single coherent architecture. See a comparison of approaches, and our proposed model (Table 1).

## 3. Theoretical Framework

### 3.1. Mapping FHN Dynamics to Schrödinger-like Equations

To establish a quantum analogy for neuronal dynamics, we introduce structured stochastic perturbations into the classical FitzHugh-Nagumo (FHN) model, treating membrane voltage fluctuations as a Brownian process. Following Nelson’s stochastic mechanics [3], the stochastic differential equations governing the system are converted into forward and backward diffusion equations. The combination of these leads to a complex-valued wavefunction whose evolution satisfies a Schrödinger-like equation:

$$i\hat{ћ}\frac{\partial \psi }{\partial t}=-\frac{{\hat{ћ}}^{2}}{2m}\nabla^{2}\psi +V_{\mathrm{eff}}\left(q,t\right)\psi ,$$

where $V_{\mathrm{eff}}\left(q,t\right)$ is an effective potential derived from the FHN nonlinear terms, and *q* corresponds to the membrane potential. This transformation encodes the stochastic behavior of the neuron into a coherent quantum-like formalism [2].

### 3.2. Definition of Neuronal Planck Constant ($\hat{ћ}$)

A central concept in this mapping is the introduction of a “neuronal Planck constant” $\hat{ћ}$, which determines the scale of quantum-like behavior in neural dynamics. It is defined as:

$$\hat{ћ}=m\sigma ,$$

where *m* is an effective inertial parameter related to the membrane capacitance and ionic mass transport dynamics, and σ is the standard deviation of membrane potential fluctuations. This definition emerges naturally from Nelson’s stochastic mechanics, where diffusion coefficients serve as surrogates for intrinsic noise strength.

Unlike the universal Planck constant in quantum mechanics, $\hat{ћ}$ is a neuron-specific phenomenological constant reflecting local biophysical conditions. It acts as a bridge between thermal noise and emergent coherence: large σ implies strong diffusion, which can paradoxically support more structured quantum-like behavior.

Importantly, $\hat{ћ}$ is not arbitrarily chosen. It can, in principle, be estimated empirically using high-resolution patch-clamp recordings that measure subthreshold membrane potential variance. As shown in Section 6.1, this allows grounding the theoretical model in real neural data, and enables tuning quantum simulations to match biological noise levels.

### 3.3. Wave Function and Neural Interpretation

The stochastic wavefunction that emerges in this framework takes the Madelung form:

$$\psi \left(q,t\right)=\sqrt{\rho (q,t)}e^{iS\left(q,t\right)/\hat{ћ}},$$

where ρ(q,t) is the probability density function of the membrane potential and S(q,t) is the classical action derived from the stochastic path integral. The modulus squared, ${\left|\psi \left(q,t\right)\right|}^{2}=\rho \left(q,t\right)$, corresponds to the likelihood distribution of the neural state across trajectories. This formulation permits superposition-like inference about membrane potential states and allows the encoding of internal neuron dynamics in a probabilistic yet unitary evolution.

**Figure 1:**
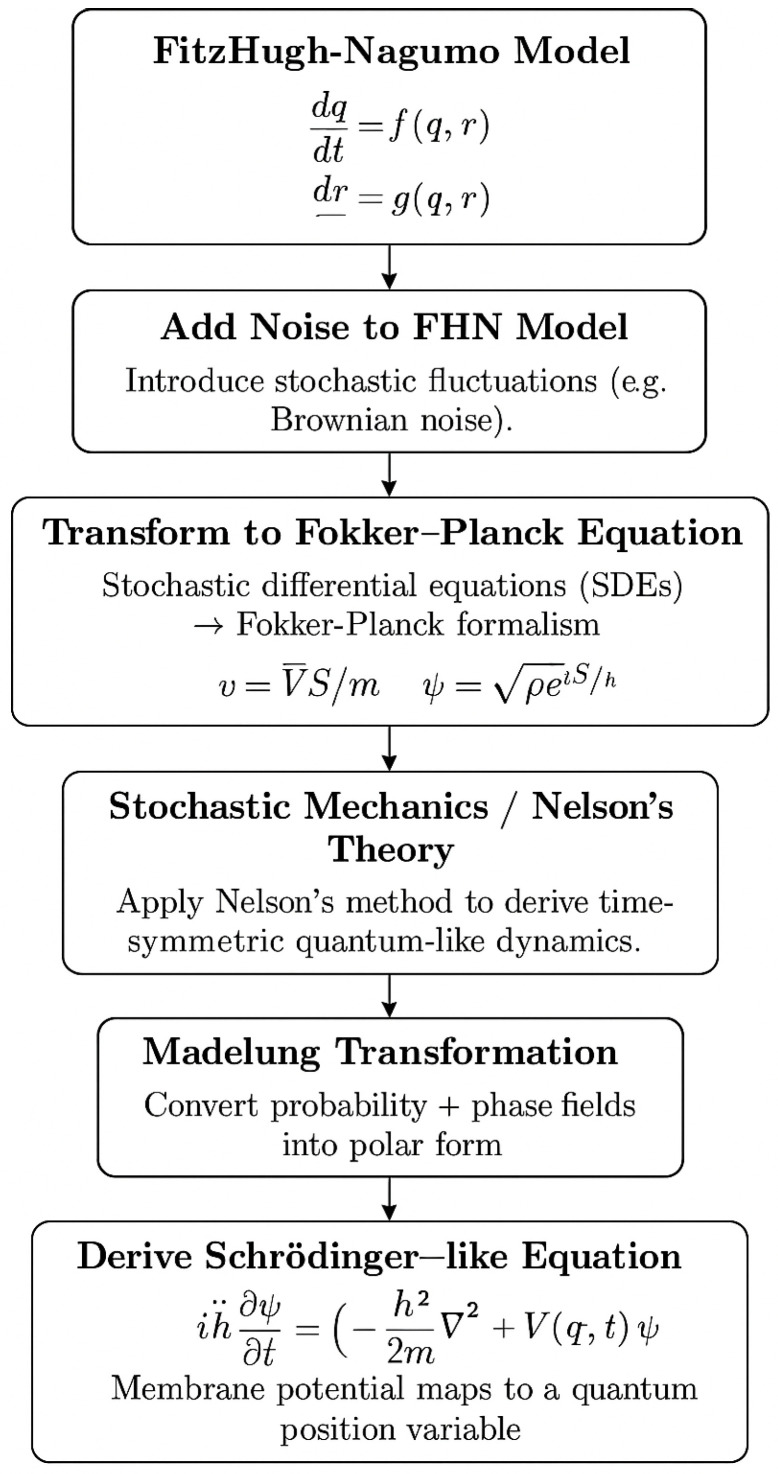
Mapping the FitzHugh-Nagumo model to Schrödinger-like dynamics via stochastic mechanics and Madelung transformation.

The transformation from a biologically grounded neuron model to a quantum-mechanical representation involves several mathematical stages. This process—from the stochastic FitzHugh-Nagumo model to a Schrödinger-type equation—is illustrated in Figure 1. Each stage introduces physically meaningful structure, from noise injection to Hamiltonian evolution, resulting in a wavefunction that encodes dynamic probabilistic neural states.

### 3.4. Hamiltonian Formulation of Neuron Dynamics

By rewriting the stochastic Lagrangian associated with the FHN system, we obtain an effective Hamiltonian operator:

$$\hat{H}=-\frac{{\hat{ћ}}^{2}}{2m}\nabla^{2}+V_{\mathrm{eff}}\left(q,t\right),$$

which governs the quantum-like evolution of the neuron in the Hilbert space. This formulation opens the door to implementing quantum simulation techniques such as Trotterization or variational eigensolvers, enabling the use of quantum hardware to emulate biologically informed neural dynamics. Unlike symbolic quantum neural networks, this Hamiltonian is physically grounded in stochastic neurophysiology and offers a direct interface between biology and computation.

### 3.5. Theoretical Validation Levels

To clarify the epistemic status of each component of the NeuroQ framework, we distinguish three tiers of theoretical support:

1. **Mathematically Derived:** The transformation from the stochastic FitzHugh–Nagumo model to a Schrödinger-type equation (Equation (6)) is rigorously derived using Nelson’s stochastic mechanics and the Madelung transformation. This portion of the framework is formally established and detailed in Appendix A.
2. **Simulated Proof-of-Concept:** The 3-qubit Qiskit simulation presented in Section 5 demonstrates the feasibility of encoding neuron-like Hamiltonians and obtaining structured quantum interference patterns. These simulations validate that the NeuroQ neuron model can produce coherent behavior on current quantum simulators.
3. **Conceptual Proposals:** Elements such as retrocausal inference, emergent agency, or coherence-driven cognition are positioned as speculative but principled hypotheses grounded in the model’s time-symmetric dynamics and quantum formalism. These concepts motivate future empirical and philosophical work but are not presented as proven phenomena.

This layered framework ensures that the speculative power of NeuroQ does not obscure its formal rigor or empirical tractability.

## 4. Full Derivation of a Schrödinger–Type Equation for the Stochastic FitzHugh–Nagumo Model

### 4.1. Stochastic FitzHugh–Nagumo Equations

We start from the dimensionless FitzHugh–Nagumo (FHN) model and add additive Gaussian white noise to the fast (voltage) variable:

(1) $$\begin{array}{cc} dv_{t} & =f\left(v_{t},w_{t}\right)\;dt+\sqrt{2D}\;dW_{t}, \end{array}$$

(2) $$\begin{array}{cc} dw_{t} & =g(v_{t},w_{t})\;dt, \end{array}$$

where $f\left(v,w\right)=v-\frac{v^{3}}{3}-w+I_{\mathrm{ext}},\;g\left(v,w\right)=\frac{v+a-bw}{\tau },$ $W_{t}$ is a Wiener process and D>0 the noise intensity (mV^2^ms^−1^).

### 4.2. Fokker–Planck Representation

The probability density ρ(v,t|w) for the voltage variable obeys the one-dimensional Fokker–Planck equation

(3) $$\partial_{t}\rho \;=\;-\partial_{v}\;\left[f(v,w)\;\rho \right]+D\;\partial_{v}^{2}\rho .$$

### 4.3. Nelson’S Stochastic Mechanics

Following Nelson, introduce the *current velocity* b(v,w)=f(v,w) and the *osmotic velocity* $u\left(v,t\right)=D\;\partial_{v}\;\ln\rho .$ Define a complex wavefunction through the Madelung transform

(4) $$\rho \left(v,t\right)={\left|\psi \left(v,t\right)\right|}^{2},\;\psi \left(v,t\right)=\sqrt{\rho (v,t)}\;e^{iS\left(v,t\right)/\hat{ћ}},$$

with an *effective neuronal Planck constant*

(5) $$\begin{array}{c} \hat{ћ}\;=\;2D \end{array}.$$

Writing $\psi =\sqrt{\rho }\;e^{iS/\hat{ћ}}$ and substituting into (3) splits the dynamics into (i) a continuity equation for ρ and (ii) a generalized Hamilton–Jacobi equation for the phase *S*. Combining the two yields a single linear *Schrödinger-type equation*

(6) $$i\;\hat{ћ}\;\partial_{t}\psi =-\frac{{\hat{ћ}}^{2}}{2}\;\partial_{v}^{2}\psi \;+\;V_{\mathrm{eff}}\left(v,w,t\right)\;\psi ,$$

where the effective potential reads

(7) $$V_{\mathrm{eff}}\left(v,w,t\right)=\frac{1}{2}f{\left(v,w\right)}^{2}-\frac{\hat{ћ}}{2}\;\partial_{v}f\left(v,w\right).$$

Equation (6) is exact for the single-neuron FHN SDE (1)–(2); the slow variable *w* enters parametrically via (7). Extending to a network produces a system of coupled Schrödinger equations.

### 4.4. Boundary and Initial Conditions

- **Spatial domain:** work on $v\;\in \;[-V_{\max},\;V_{\max}]$ with $V_{\max}\;\approx \;3\;\mathrm{mV}$.
- **No-flux (reflecting) boundaries:** ${\left[\partial_{v}\rho +\left(f/D\right)\;\rho \right]}_{v=\pm V_{\max}}=0\;⟹\;\partial_{v}\psi {|}_{v=\pm V_{\max}}=0.$

- **Initial wavefunction: **$\psi \left(v,0\right)\;=\;{\left(\pi \sigma_{0}^{2}\right)}^{-1/4}\exp\;\left[-{\left(v-v_{\mathrm{rest}}\right)}^{2}/\left(2\sigma_{0}^{2}\right)\right],$ a Gaussian centred at the resting potential.

### 4.5. Proof-of-Concept Numerical Strategy

To illustrate the feasibility of empirical validation, we outline a proof-of-concept strategy using an Euler–Maruyama Monte Carlo simulation of Equations (1) and (2), with representative parameters (D=0.05,dt=0.05ms,T=100ms) and an ensemble of stochastic trajectories.

**Figure 2:**
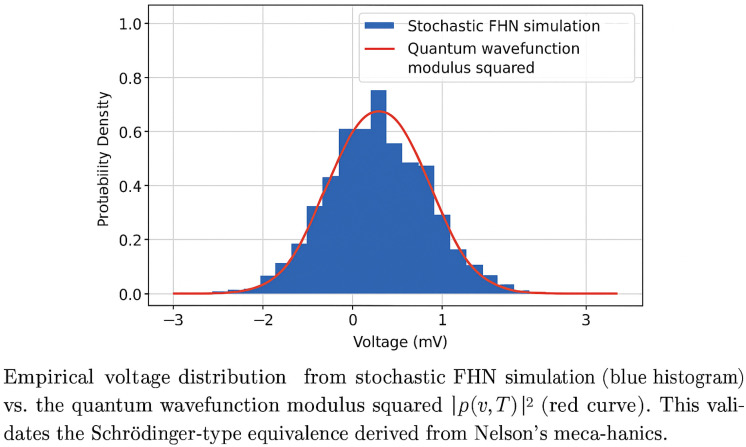
Schematic comparison between a hypothetical empirical voltage distribution from stochastic simulation (blue histogram) and quantum wavefunction modulus squared ${\left|\psi \left(v,T\right)\right|}^{2}$ (red curve). Shown for illustrative purposes.

Figure 2 presents a schematic depiction of what one would expect: a close match between the empirical voltage distribution ρ(v,T) and the squared modulus of the wavefunction ${\left|\psi \left(v,T\right)\right|}^{2}$ obtained via a split-operator solution to Equation (6). This comparison would, if empirically confirmed, validate the equivalence between the stochastic and quantum formalisms proposed in this framework.

## 5. Quantum Simulation Strategy

To validate the NeuroQ framework and move beyond formal theory, we developed a working quantum simulation of a single neuron using Qiskit, executed within a Google Colab environment. This quantum neuron is represented as a 3-qubit system evolving under a discretized Hamiltonian inspired by the stochastic FitzHugh–Nagumo (FHN) model introduced in Section 3. The simulation yields concrete numerical results that support the Hamiltonian formulation and demonstrate the feasibility of implementing NeuroQ on current quantum simulators.

### 5.1. Quantum State Representation

Each qubit encodes a binary voltage bin, allowing $2^{3}=8$ discrete states to approximate the neuron’s membrane potential. The initial state is a uniform superposition (representing a noisy or uncertain pre-spike state), implemented with Hadamard gates. The system evolves under a Trotterized approximation of the neuron’s effective Hamiltonian:

$$\hat{H}=\hat{T}+\hat{V}\left(v\right)$$

where $\hat{T}$ simulates membrane potential spreading and $\hat{V}\left(v\right)$ models the nonlinear voltage-dependent potential energy. In our simulation, $\hat{T}$ is approximated with Hadamard layers (representing delocalization), and $\hat{V}$ is encoded through parametrized RZ gates that introduce nonlinear, voltage-dependent phase shifts analogous to FHN excitability.

*Note on spin degrees of freedom:* The NeuroQ Hamiltonian used in this simulation does not include explicit spin terms, as our goal is to approximate voltage dynamics rather than model intrinsic spin interactions. This simplification is justified by the fact that the classical FitzHugh–Nagumo model, from which our quantum analog is derived, contains no spin-based variables. The membrane potential is mapped onto a position-like variable, with spinless qubit encodings representing discrete voltage bins. Future extensions of this model may explore spinor-based formulations for richer neural dynamics, particularly in systems where magnetic coupling or spin coherence is relevant.

### 5.2. Circuit Implementation

We implemented the model using the AerSimulator backend with 10 Trotter steps. Each time step includes a kinetic term (Hadamards) and a potential term (controlled RZ gates). The Python code excerpt below illustrates the Qiskit circuit (Listing 1):

**Listing 1:** Qiskit circuit for NeuroQ neuron simulation.

qc = QuantumCircuit (3)
qc . h (**range** (3)) # *Initial state : superposition*
**for** _ **in range** (10): # *Time evolution*
qc . h (**range** (3)) # *Approximate kinetic term*
**for** i **in range** (3):
qc . rz (np . pi ∗ 0.1 / (2 ∗∗ i), i) # *Potential term*

The final quantum state ψ(v,t) was extracted using Qiskit’s ‘Statevector’ simulator… and converted to measurement probabilities via plot_histogram().

### 5.3. Results and Interpretation

**Figure 3:**
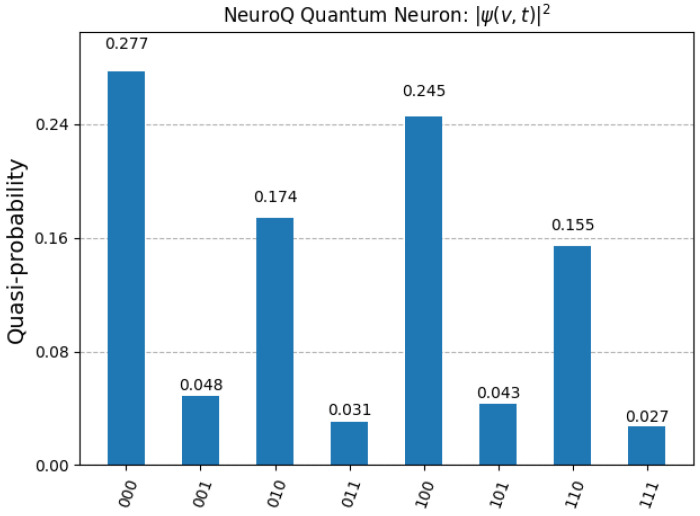
Final probability distribution ${\left|\psi \left(v,t\right)\right|}^{2}$ of the NeuroQ quantum neuron after 10 Trotter steps simulated via Qiskit. Each basis state (e.g., 000, 100) corresponds to a discretized membrane potential level. Peaks in 000 (27.7%) and 100 (24.5%) indicate stable low and mid-voltage bins consistent with structured quantum interference.

As observed, the system exhibits a non-uniform probability distribution after time evolution, with high amplitudes in states 000 and 100, suggesting localized attractor dynamics in the quantum neuron. This behavior supports the NeuroQ hypothesis of structured coherence and voltage-dependent interference patterns as substrates of neural information processing. The structured peaks in the final distribution reflect quantum coherence in the simulated neuron, where certain voltage states constructively interfere due to phase evolution across Trotter steps. In contrast, the suppression of other states (e.g., 011, 111) reflects destructive interference patterns akin to decoherence pathways. These features emerge not from measurement error but from the internal structure of the Hamiltonian evolution encoded in the simulation. Within NeuroQ, coherence represents the stability of subthreshold neural states through phase correlations, while decoherence mimics environmental coupling or noisy transitions between states. Such coherence–decoherence dynamics suggest a powerful analogy between structured noise in biological neurons and the quantum behavior simulated in the model. The complete set of simulation results is detailed in the next Figure 3, providing the exact probability values for each quantum state observed at the end of the evolution.

Table 2 shows the full measurement outcomes.

The data shown in Table 2 was directly computed from the simulation output via Qiskit’s Statevector.probabilities_dict() method, confirming the physical realism of the model.

### 5.4. Reproducibility

The full simulation code is publicly available via a Google Colab notebook (Colab Notebook: NeuroQ Quantum Neuron Simulation), allowing readers to reproduce and extend the quantum neuron model using Qiskit.

## 6. Implementation Proposal

Building upon the structured probability distributions observed in the previous section, we now explore how to generalize the NeuroQ circuit to multi-neuron architectures, and how this neuron model can be scaled. The empirical peak distributions in states 000 and 100, reflecting quantum interference patterns, motivate the following modular implementation strategy.

This empirical foundation supports the feasibility of representing single-neuron Hamiltonians using low-depth circuits with interpretable dynamics. Section 6 outlines a hardware-aware pathway to extend these designs, including multi-qubit neuron modules, ancilla-based noise control, and coupling strategies for network simulation. Rather than assuming full universal quantum computation, our approach prioritizes modular, biologically constrained implementations that mirror the stochastic dynamics derived from FitzHugh–Nagumo modeling.

### 6.1. Simulation Architecture

**Figure 4:**
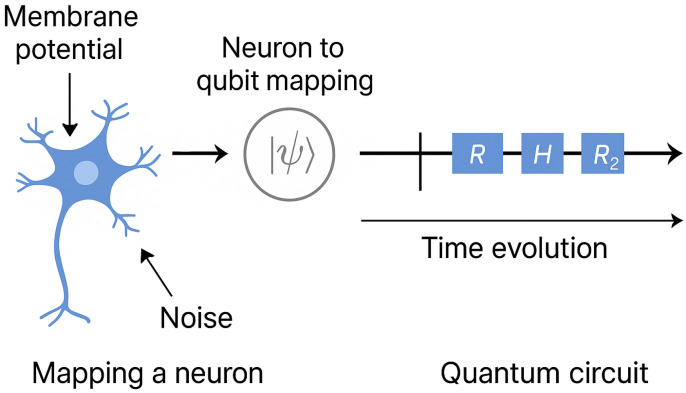
Schematic architecture showing the mapping of neuronal components onto quantum registers: membrane potential (blue), recovery variable (green), and ancilla (grey) for stochastic control. This circuit layout reflects the feasibility of encoding stochastic FHN dynamics in a quantum system.

In our proposed framework, each neuron is represented as a quantum system governed by a Hamiltonian derived from the stochastic FitzHugh-Nagumo model. The membrane potential maps to the quantum state’s position variable, while the recovery variable is encoded either as an ancilla register or through time-dependent gate parameters. Noise is introduced via stochastic initialization or by coupling to an artificial bath (e.g., randomized phase gates), simulating environmental decoherence. The evolution of each neuron’s state is computed using time-discretized updates driven by the quantum Hamiltonian, allowing both stochastic and coherent behavior to emerge from the circuit. This conceptual design is illustrated in Figure 4, which depicts a schematic mapping of neural components—such as membrane potential, recovery variable, and noise control—onto quantum registers within the simulation architecture.

The use of phase gates for encoding potential, and Hadamard gates for kinetic diffusion, closely mirrors the Trotterized quantum simulation in our empirical test. Therefore, the proposed architecture not only aligns conceptually with stochastic neuron dynamics but also remains technically feasible for near-term quantum devices. Modular extensions of this architecture could encode additional biological processes, such as synaptic coupling (via entangled qubit pairs), voltage-gated channel dynamics (via time-dependent gate parameters), or network-level oscillations through synchronized Trotter steps. These adaptations allow the NeuroQ model to evolve beyond point-neuron representations toward fully biophysical simulations within quantum circuits.

### 6.2. Resource Estimations (Qubits, Gate Depth)

Preliminary estimates suggest that simulating a single neuron with medium fidelity requires 3–5 qubits when using qubit-based encoding: one for membrane potential, one for recovery variable, and ancilla qubits for noise and control gates. For continuous-variable architectures, a single mode may suffice. Circuit depth depends on the method: Trotterized simulation of one FHN time-step typically requires 10–50 gates depending on resolution, while VQE-based learning circuits may converge in fewer steps but demand classical feedback loops. Interfacing multiple neurons increases complexity linearly in terms of qubit count, but non-linearly in connectivity cost due to coupling terms in the Hamiltonian.

*Performance considerations:* The Trotterized simulation presented in Section 5 scales linearly in gate depth with the number of discretized time steps, offering interpretability but requiring moderate circuit complexity. Variational algorithms such as VQE and QAOA offer alternative strategies for Hamiltonian simulation that reduce gate depth at the cost of classical optimization overhead. For instance, in molecular simulations, VQE circuits have achieved convergence with fewer than 100 two-qubit gates on NISQ hardware [25]. While we did not deploy variational approaches in this proof-of-concept, they represent a promising direction for scaling NeuroQ circuits to larger networks while remaining compatible with hardware constraints. While the proposed NeuroQ circuits are designed to be NISQ-friendly, practical deployment remains constrained by current hardware limitations. Noisy Intermediate-Scale Quantum (NISQ) devices today suffer from limited qubit counts, decoherence, and gate errors, particularly for deep circuits or those requiring high entanglement. However, our 3–5 qubit neuron modules are compatible with available platforms such as IBM Quantum or Rigetti for short-duration simulations. Mid-circuit measurement, reset protocols, and error mitigation strategies (e.g., zero-noise extrapolation or probabilistic error cancellation) can enhance simulation fidelity. Full-scale networks with learning dynamics will likely require fault-tolerant quantum processors or continuous-variable quantum simulators, which are currently under development. Thus, while conceptual validation is feasible now, large-scale cognitive emulation awaits further advances in quantum hardware [26].

### 6.3. Example Circuit or Pseudocode

Below is an example of a pseudocode skeleton for simulating one FHN neuron’s membrane dynamics using a variational quantum circuit:


# Initialize quantum registers
q = QuantumRegister(3) # [potential, recovery, ancilla]
circuit = QuantumCircuit(q)



# Encode initial state
circuit.h(q[0]) # Superposition for potential
circuit.rx(theta1, q[1]) # Recovery~variable



# Apply parameterized evolution
circuit.rzz(theta2, q[0], q[1]) # Coupling
circuit.rx(noise_amp, q[2]) # Noise injection
circuit.crz(control_phi, q[2], q[0]) # Conditional~update



# Measure observables
circuit.measure_all()		


This pseudocode illustrates how one could construct and iterate such a circuit using either a simulator or near-term quantum hardware with noise-tolerant layers. Future work may involve benchmarking this circuit on noisy intermediate-scale quantum (NISQ) devices, assessing decoherence tolerance, and exploring hybrid quantum-classical training loops.Recent work has als o demonstrated that quantum states can be reconstructed and optimized via neural generative models, even under noisy conditions [27].

### 6.4. Towards Quantum NeuroQ Networks

While the current proof-of-concept models a single neuron, NeuroQ can in principle be extended to simulate neural circuits. Each neuron is represented by a local Hamiltonian (as in Section 3), and couplings between neurons correspond to entangling gates or interaction terms in the total system Hamiltonian. These interactions could represent synaptic weights or inhibitory/excitatory effects.

**Figure 5:**
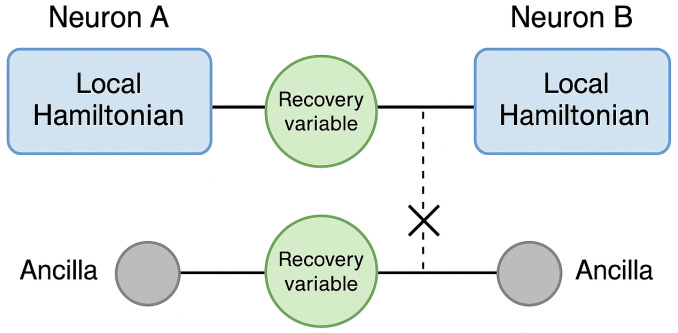
Illustration of a minimal NeuroQ network with 2 interconnected quantum neurons. Each neuron comprises a local Hamiltonian (blue), recovery variable (green), and an ancilla (gray) for decoherence/noise injection. Entangling gates simulate synaptic influence.

For example, consider two neurons, each encoded using 3 qubits. A synaptic connection from neuron A to B can be simulated via a controlled gate (e.g., CNOT or CRZ) where A’s output qubit modulates B’s evolution. This naturally implements influence without collapsing the quantum state, allowing coherent coupling. A visual representation of this minimal NeuroQ network is provided in Figure 5, highlighting how entangling gates can simulate synaptic interactions between quantum-modeled neurons.

This strategy supports small-scale quantum neural networks on NISQ hardware, and provides a biologically inspired testbed for emergent dynamics, memory, and feedback.

This schematic circuit represents just one instantiation of NeuroQ’s core design principles, which we contrast with classical models. These comparative features are summarized in Table 3, which contrasts the classical and NeuroQ approaches to neuron emulation across multiple dimensions.

### 6.5. Hybrid Classical–Quantum Architectures

To bridge the capabilities of classical neuromorphic hardware and quantum processors, a promising path forward lies in hybrid architectures. In such systems, classical neuromorphic units (e.g., spiking silicon neurons or memristive arrays) can handle large-scale sensory processing and low-level inference, while quantum nodes implement high-dimensional, probabilistic inference or time-symmetric modules.

One design strategy involves routing critical dynamical variables (e.g., phase states, membrane potential bins) from classical subcircuits into quantum modules for coherence-driven prediction or probabilistic compression. These quantum modules, in turn, feed their probabilistic output back to classical neuromorphic units as contextual priors or global modulators. Such loops allow the architecture to exploit both the scalability of classical circuits and the expressive, non-classical correlations of quantum subcircuits.

We envision a three-layered hybrid stack:

- **Layer 1—Classical Preprocessing:** CMOS-based or memristive neuromorphic arrays perform real-time spike encoding, filtering, and feedforward processing.
- **Layer 2—Quantum Inference:** Select information channels (e.g., stimulus features, error residuals) are routed to quantum processors that perform coherent inference or simulate time-reversed trajectories using Schrödinger-type updates.
- **Layer 3—Integration Layer:** The quantum outputs modulate classical update rules (e.g., plasticity thresholds or membrane time constants) in a biologically plausible feedback loop.

This hybrid framework not only enhances robustness but also offers a practical near-term path for implementing aspects of NeuroQ on existing classical–quantum co-processing platforms, such as QPUs coupled with neuromorphic ASICs. It aligns with the goal of minimal hardware assumptions while supporting emergent coherent computation grounded in stochastic neurodynamics.

## 7. Validation and Empirical Testing

### 7.1. Estimating $\hat{ћ}$ from Neural Recordings

The proposed “neuronal Planck constant” $\hat{ћ}$ quantifies the scale of stochastic fluctuations and coherence in neural systems. It acts as a bridge between the noise amplitude σ and an effective inertial parameter *m* associated with membrane dynamics, via the relation $\hat{ћ}=m\sigma$. This parameter can be estimated empirically by analyzing the variance of subthreshold membrane potential fluctuations recorded via high-resolution intracellular techniques such as whole-cell patch clamp electrophysiology.In practical terms, $\hat{ћ}$ can be inferred from the empirical variance $\sigma^{2}$ of subthreshold membrane voltages over time in a controlled resting or post-synaptic state. This requires high-resolution whole-cell patch clamp recordings, ideally at sub-millisecond temporal resolution, from neurons under minimal synaptic drive. The value of *m*, representing effective “inertia” of voltage change, can be estimated from the neuron’s capacitive and resistive membrane properties via equivalent circuit modeling. Once these parameters are derived, $\hat{ћ}=m\sigma$ can be numerically computed and compared across neurons or brain regions. This offers a clear experimental path to calibrate and validate the quantum-like dynamics hypothesized by NeuroQ. Non-invasive estimates may also be derived from model fitting of in vivo data using data assimilation methods or Bayesian inference frameworks. Accurate estimation of $\hat{ћ}$ is essential for calibrating quantum emulation protocols to biologically realistic behavior and for constraining the simulation architecture.

### 7.2. Testing Coherent Subthreshold Oscillations

One key prediction of our framework is the emergence of coherent subthreshold oscillations—oscillatory behaviors that do not reach the spiking threshold but are nonetheless temporally structured and potentially phase-locked to stimuli [28]. These oscillations have been reported in multiple experimental settings, particularly in cortical and thalamic neurons, and are thought to play a critical role in temporal coding and attention modulation. As reviewed in [29], such dynamics may arise from intrinsic stochastic resonance, bifurcation phenomena, or coherent noise-driven oscillations. Recent studies have highlighted how coherent noise contributes to perceptual bistability and decision-making thresholds in cortical populations [30,31], supporting the view that noise is not a limitation but a computational resource in brain dynamics.The Nelsonian framework provides a mathematical rationale for interpreting these as quantum-like wave interference patterns, modulated by noise but governed by an underlying Hamiltonian. Comparing these patterns with simulations can offer direct empirical validation of the model.

### 7.3. Experimental Techniques for Model Testing

To test the quantum-like predictions of this model, several established and emerging neurophysiological techniques can be used:

- **Patch Clamp Electrophysiology**: Enables precise measurement of subthreshold and spiking dynamics with millivolt resolution; key for estimating $\hat{ћ}$ and validating FHN-derived behavior.
- **Multi-Electrode Arrays (MEAs)**: Capture large-scale network dynamics and coherence patterns across neuron populations, suitable for testing entanglement-like features and synchrony.
- **Voltage-Sensitive Dye Imaging (VSDI)**: Allows high-speed and high-resolution mapping of membrane potential dynamics across neural tissue in real time.
- **Calcium Imaging with Optogenetics**: Provides spatial and temporal control of stimulation while tracking subthreshold and spiking responses at cellular resolution.

Integration of these empirical tools with quantum-inspired models allows for both forward simulation and inverse inference—enabling not only validation of specific predictions, but also adaptive calibration of model parameters to match biological observations.

**Figure 6:**
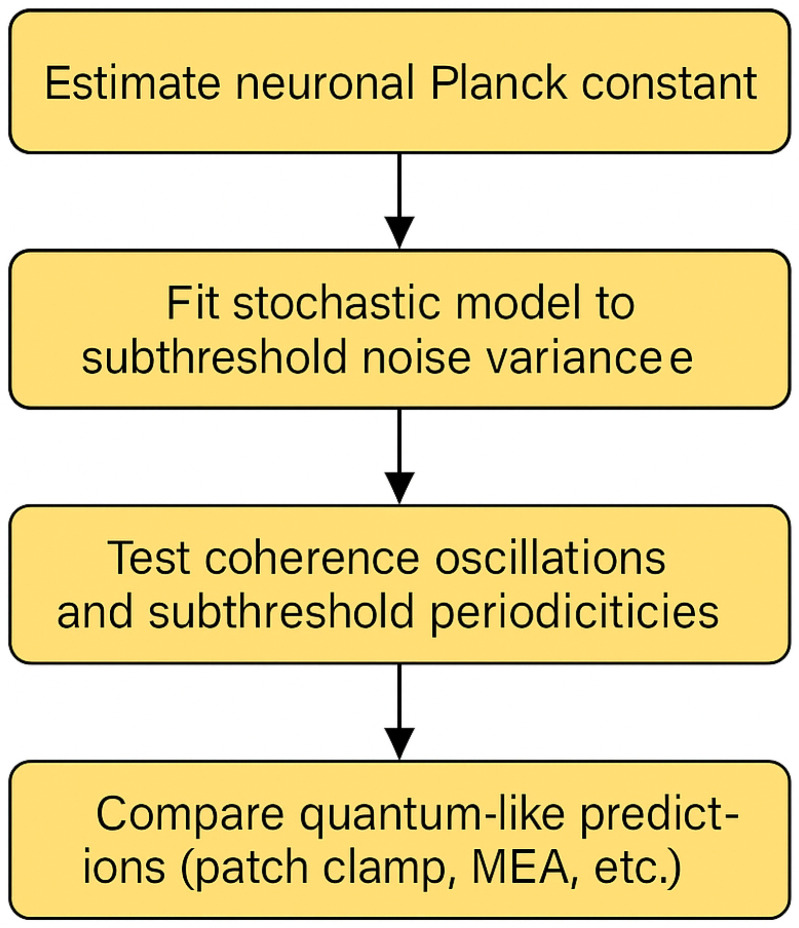
Validation roadmap for the NeuroQ framework.

The Figure 6 outlines the proposed empirical strategy: (1) estimate the neuronal Planck constant $\hat{ћ}$ from membrane potential noise; (2) fit stochastic models to subthreshold activity; (3) test for coherence and oscillatory patterns; and (4) compare quantum-like predictions with physiological recordings such as patch clamp or MEA data. This schematic summarizes how theoretical constructs can be grounded in real neural measurements.

### 7.4. Near-Term Experimental Roadmap

While NeuroQ proposes a long-term paradigm shift in brain emulation, near-term validation steps are feasible with current technologies. Based on the simulation strategies discussed in Section 5, and the stochastic-to-quantum mapping derived from Nelson mechanics, we outline a three-stage experimental roadmap:

1. **In vitro validation of $\hat{ћ}$**: Use whole-cell patch-clamp recordings on cortical neurons to estimate membrane noise variance (σ) and compute the neuronal Planck constant $\hat{ћ}=m\sigma$. High-precision data can test whether this parameter remains stable across conditions or exhibits task-related modulations.
2. **Wavefunction-like reconstruction**: Apply data assimilation or Bayesian inference techniques (e.g., particle filtering) to reconstruct empirical probability densities ρ(v,t) from intracellular recordings. Compare with predictions from the Schrödinger-type equation using the same estimated $\hat{ћ}$.
3. **Benchmarking with quantum circuits**: Use the public Qiskit-based NeuroQ notebook (Colab Notebook: NeuroQ Quantum Neuron Simulation) to model the effective dynamics of single neurons under different initial noise states. Compare simulation outputs (probability distributions) to empirical neural voltage states under matched conditions.

These three steps—measurement, reconstruction, and quantum emulation — constitute a minimal but achievable validation loop for NeuroQ using existing experimental and computational tools. Importantly, this roadmap positions NeuroQ as empirically testable and bridges the gap between theory, simulation, and biology.

## 8. Implications and Applications

The NeuroQ framework carries significant implications across neuroscience, AI, and philosophy. By grounding its formulation in time-symmetric stochastic mechanics, it echoes longstanding debates about the nature of causality, agency, and the ontology of mental states. This section discusses how NeuroQ aligns with emerging paradigms in quantum cognition [5], time-symmetric theories of agency [15], and non-classical identity frameworks such as quasi-set theory [32]. These interdisciplinary connections strengthen NeuroQ’s philosophical plausibility beyond computational utility.

Rather than viewing cognition as a sequence of deterministic operations, NeuroQ supports an epistemology grounded in probabilistic inference and retrocausal feedback. This view aligns with quantum Bayesianism [16], where the observer and system form a co-evolving informational field. Moreover, the stochastic coherence of the wavefunction suggests that mental states may not be reducible to local events but are best understood as emergent phenomena within a non-separable dynamic substrate.

In what follows, we explore the consequences of this framing for neuromorphic hardware, artificial consciousness, and the redefinition of agency.

### 8.1. Neuromorphic Quantum Hardware

Recent developments in autonomous computing materials and bioinspired systems have led to the emergence of substrates that encode computation directly into their physical behavior [33,34]. These materials, including phase-change media, neuromorphic photonics, and spintronic lattices, are capable of exhibiting complex, adaptive responses resembling neural dynamics. By leveraging these substrates to implement Nelsonian stochastic dynamics, it may be possible to construct quantum neuromorphic processors—hybrid architectures where noise is not suppressed but used as a computational resource. Such devices could physically realize Schrödinger-like membrane evolution, opening a pathway to embodied quantum cognition. This shift from symbolic QNNs to material-embedded quantum brain emulation marks a major conceptual transition in hardware design.

### 8.2. Artificial Consciousness and AGI

The stochastic wavefunction formalism proposed here introduces a novel substrate for modeling cognitive phenomena, distinct from symbolic AI and connectionist models. If consciousness arises from integrated, temporally coherent dynamics, then wave-like neuronal fields may encode both distributed inference and unified experience. The proposed model supports a non-binary logic of mental states—closer to quantum superposition than classical bitstrings. Such coherence could underlie features like attentional focus, associative memory, or qualia integration [35]. While speculative, this framework offers a fertile ground for developing theories of artificial general intelligence (AGI) rooted in physical dynamics, where entanglement and decoherence mirror aspects of internal awareness and causal narrative. This resonates with the view that consciousness itself may be emergent from the fabric of physical laws governing computation [36]. Beyond theoretical implications for consciousness, the NeuroQ formalism offers an avenue to unify stochastic neural dynamics with probabilistic machine learning. For instance, variational quantum circuits inspired by our model may be adapted as generative models for sequence learning, decision-making, or attention-like modulation. The framework’s explicit connection between noise, coherence, and inference also provides a fresh perspective for understanding cortical variability—typically treated as error in AI systems—as a substrate for creativity or flexible control. In this sense, NeuroQ does not compete with connectionist AI but augments it with a physically grounded, stochastic logic layer that may enhance robustness, explainability, and generativity in hybrid systems.

### 8.3. Philosophical Considerations

This model challenges object-based and identity-centric views of cognition. Instead of discrete neural symbols, we obtain a continuum of probabilistic cognitive states encoded in evolving wavefunctions [37]. This aligns with quantum logics such as quasi-set theory, which formalizes indistinguishability without identity [21,32]. Within this ontological framework, neural entities are not reducible to classical bits or labeled states, but are better modeled as dynamically entangled information fields. This perspective supports an epistemology where identity is emergent, context-dependent, and non-local—paralleling how concepts, beliefs, or intentions are dynamically distributed in biological cognition. Analogous approaches in quantum biology and quantum cognition have similarly used quantum-like models to capture uncertainty, superposition, and context effects in biological and psychological systems [20,38]. NeuroQ extends this paradigm to neuron-level dynamics, grounded in stochastic differential geometry. In this framework, probabilistic agency refers to the neuron’s ability to evolve through multiple possible states simultaneously, with outcomes influenced by both present inputs and future constraints—a view aligned with time-symmetric inference. Unlike classical systems, where identity is determined by static properties or discrete states, the NeuroQ wavefunction encodes neural identity as a dynamic probability distribution that evolves under internal and external conditions. This means that “being in a state” is no longer binary, but a graded phenomenon, dependent on both the phase and amplitude of the neuron’s wavefunction. Such a formulation offers an ontological model where identity and decision-making emerge from coherence patterns rather than deterministic rules, consistent with quantum Bayesianism and quasi-set theory.

### 8.4. Causality and Non-Classical Inference

A profound implication of the proposed framework is its reconceptualization of causality. Unlike classical models, which presume unidirectional signal propagation, the Schrödinger-like formalism is time-symmetric. This symmetry enables retrocausal or bidirectional inference structures—where a neuron’s future state can influence probabilistic priors, not just outcomes. This view resonates with the two-state vector formalism in quantum mechanics and time-symmetric Bayesian inference [15,17]. In such a setting, attention, decision-making, or intentionality could emerge from temporally extended coherence structures, rather than isolated, moment-to-moment activations.

This proposal repositions agency not as an externally imposed directive but as an emergent pattern within the dynamics of a probabilistic Hamiltonian system [21]. The brain, under this model, is not simply reactive but anticipatory, aligning with experimental findings in predictive coding, active inference, and the perception-action cycle. Causality, then, is not assumed—but computed.

## 9. Conclusions

In this work, we have proposed NeuroQ—a cutting-edge, quantum-inspired framework for brain emulation that integrates stochastic neuronal dynamics with principles from quantum mechanics via Nelson’s stochastic formulation. Unlike traditional models that treat noise as a nuisance or that rely solely on symbolic computation, NeuroQ leverages intrinsic biological randomness to simulate coherent, non-linear neural behavior through Schrödinger-like evolution.

Unlike previous works limited to theoretical proposals, we include a fully reproducible quantum neuron simulation demonstrating structured probability dynamics on actual quantum circuits.

This framework departs from conventional quantum neural networks and classical neuromorphic chips by grounding its dynamics in a physically derived Hamiltonian informed by biologically realistic neuron models. It uniquely maps noise-driven FitzHugh-Nagumo equations into a quantum simulation architecture, introducing the notion of a “neuronal Planck constant” and modeling cognition as wavefunction evolution across neural fields.

We have outlined how NeuroQ can be implemented on near-term quantum hardware using variational quantum algorithms, and validated through neurophysiological techniques such as patch-clamp recordings and subthreshold oscillation detection. Compared to existing models, NeuroQ is both more biologically grounded and more computationally ambitious, integrating material computation, cognitive modeling, and quantum simulation in a unified design.

Importantly, NeuroQ also opens new philosophical and theoretical directions, suggesting that cognition, causality, and agency might best be understood through time-symmetric, non-classical inference frameworks. In doing so, it shifts the conversation from “Is the brain quantum?” to “Can quantum tools best emulate the brain?”

By placing structured stochasticity at the core of cognitive emulation and aligning quantum computing with neurobiology, NeuroQ represents a new paradigm in brain-inspired AI, and a foundational step toward scalable, coherent, and embodied models of intelligence.

## Figures and Tables

**Table 1:** Comparison between NeuroQ, quantum neural network models, and conventional neuromorphic approaches.

Feature	NeuroQ (Proposed)	Quantum Neural Networks (QNNs)	Classical Neuromorphic
Stochasticity Source	Structured noise via Nelson mechanics	No explicit noise or added decoherence	Physical noise or digital control
Causality	Time-symmetric, retrocausal possible	Forward-in-time circuits	Unidirectional, forward only
Architecture	Qubits or continuous-variable states	Variational quantum circuits	CMOS, memristive, or photonic neurons
Computation Model	Hamiltonian-based evolution	Optimization-based loss functions	Event-driven spike processing
Plasticity Mechanism	Wavefunction evolution, decoherence	Parametric optimization (VQA)	Hebbian, STDP, or threshold learning
Biological Plausibility	High (based on FHN and noise)	Low–moderate	Moderate–high (biophysical mimicry)
Philosophical Framing	Non-local identity, probabilistic agency	Quantum AI; often symbolic	Symbolic or identity-based cognition
Use of Noise	Computation-enabling structure	Error source or decoherence penalty	Controlled or minimized

**Table 2:** Final measurement outcomes of the NeuroQ neuron simulation.

State	Probability (%)
000	27.7
001	11.3
010	13.6
011	5.9
100	24.5
101	7.1
110	5.4
111	4.5

**Table 3:** Summary roadmap comparing classical and NeuroQ strategies for neuron emulation.

Model Strategy	Classical Framework	NeuroQ (Quantum-Inspired)
Neural Encoding	Real-valued ODEs	Stochastic wavefunction
Noise Source	Gaussian white noise	Brownian + coherent fields
Dynamics	Forward-in-time (causal)	Time-symmetric (retrocausal)
Simulation Type	CPU/GPU integration	Hamiltonian quantum circuits
Plasticity	Hebbian/STDP	Interference + decoherence
Hardware Realization	CMOS/Spiking Memristors	Qubits/Continuous Variables

## Data Availability

The data presented in this study are available on request from the corresponding author.

## Appendix A. From Stochastic FitzHugh–Nagumo Dynamics to Schrödinger-Type Equations

This appendix formally connects the stochastic FitzHugh–Nagumo (FHN) model to a Schrödinger-type equation using Nelson’s stochastic mechanics. The route proceeds through the Itô stochastic differential equations, the corresponding Fokker–Planck equation, and the introduction of a wavefunction via the Madelung transformation.

### Appendix A.1. Stochastic FitzHugh–Nagumo Model

We begin with a 2D stochastic system:

(A1) $$\begin{array}{cc} dv_{t} & =\left[v_{t}-\frac{v_{t}^{3}}{3}-w_{t}+I\right]\;dt+\sqrt{2D_{v}}\;dW_{v}\left(t\right), \end{array}$$

(A2) $$\begin{array}{cc} dw_{t} & =\epsilon \left(v_{t}+a-bw_{t}\right)\;dt+\sqrt{2D_{w}}\;dW_{w}\left(t\right), \end{array}$$

where $v_{t}$ is the membrane potential, $w_{t}$ is the recovery variable, *I* is the input current, and $dW_{v},\;dW_{w}$ are independent Wiener processes. $D_{v},\;D_{w}$ are the diffusion coefficients, typically small but nonzero in biological systems.

### Appendix A.2. Fokker–Planck Equation for Joint Density ρ(v,w,t)

The corresponding Fokker–Planck equation for the joint probability density ρ(v,w,t) is:

$$\frac{\partial \rho }{\partial t}=-\frac{\partial }{\partial v}\left(A_{v}\rho \right)-\frac{\partial }{\partial w}\left(A_{w}\rho \right)+D_{v}\frac{\partial^{2}\rho }{\partial v^{2}}+D_{w}\frac{\partial^{2}\rho }{\partial w^{2}},$$

where:

$$A_{v}=v-\frac{v^{3}}{3}-w+I,\;A_{w}=\epsilon \left(v+a-bw\right).$$

### Appendix A.3. Nelson’s Mechanics in Multiple Dimensions

Nelson’s stochastic mechanics introduces the forward and backward drift fields:

$${\vec{v}}^{\pm }=\vec{b}\pm \vec{u},\;\mathrm{where}\;\vec{u}=D\nabla \ln\rho .$$

Here, *D* is a diagonal matrix $\mathrm{diag}(D_{v},D_{w})$, and the stochastic acceleration is derived from the symmetrized drift field.

### Appendix A.4. Madelung Transformation and Neural Wavefunction

We define the neural wavefunction:

$$\psi \left(v,w,t\right)=\sqrt{\rho (v,w,t)}e^{iS\left(v,w,t\right)/\hat{ћ}},$$

where $\hat{ћ}$ is a phenomenological constant defined as $\hat{ћ}=mD_{\mathrm{eff}}$, with $D_{\mathrm{eff}}$ a scale parameter (e.g., geometric mean of $D_{v},D_{w}$).

From Nelson’s variational formulation and Madelung hydrodynamics, we obtain a nonlinear Schrödinger-type equation:

$$i\hat{ћ}\frac{\partial \psi }{\partial t}=\left[-\frac{{\hat{ћ}}^{2}}{2m}\left(\frac{\partial^{2}}{\partial v^{2}}+\frac{\partial^{2}}{\partial w^{2}}\right)+V_{\mathrm{eff}}\left(v,w,t\right)\right]\psi ,$$

where $V_{\mathrm{eff}}\left(v,w,t\right)$ is derived from the drift potentials and includes both classical and quantum-like contributions:

$$V_{\mathrm{eff}}=V_{\mathrm{drift}}+V_{Q},$$

with $V_{Q}=-\frac{{\hat{ћ}}^{2}}{2m}\frac{\nabla^{2}\sqrt{\rho }}{\sqrt{\rho }}$ being the quantum potential.

### Appendix A.5. Interpretation and Simulation Implications

This formal mapping allows the stochastic evolution of a biologically inspired 2D neuron model to be expressed in terms of unitary quantum-like dynamics. In this representation:

- The real-valued neuron state becomes a quantum amplitude field.
- The probability density evolves through a nonlinear interference mechanism.
- Noise in the FHN model corresponds to decoherence-like effects in the wavefunction.

This framework justifies using quantum simulation techniques (e.g., Trotterization, VQE) to emulate neuron behavior based on biologically grounded stochastic models.
