# Correction: Vallverdú, J.; Rius, G. NeuroQ: Quantum-Inspired Brain Emulation. *Biomimetics* 2025, *10*, 516

**DOI:** 10.3390/biomimetics10120797

**Published:** 2025-11-25

**Authors:** Jordi Vallverdú, Gemma Rius

**Affiliations:** 1 Philosophy Department, ICREA-UAB, Bellaterra, 08193 Barcelona, Spain; 2 Institut de Microelectrònica de Barcelona, IMB-CNM (CSIC), Bellaterra, 08193 Barcelona, Spain; gemma.rius@csic.es

There was an error in the original publication [1]; certain explanatory details and attributions required clarification.

A correction has been made to the following:

**Section 3.2 Definition of Neuronal Planck Constant (** $\hat{ћ}$ **), first paragraph:**

**Corrected paragraph:**

We define the neuronal analogue of Planck’s constant as

$$\hat{ћ}=m\sigma ,$$

following Ghose and Pinotsis [2]. Here *m* is an effective inertial parameter related to the membrane capacitance and ionic mass transport dynamics, and *σ* is the standard deviation of membrane potential fluctuations. This definition emerges naturally from Nelson’s stochastic mechanics, where diffusion coefficients serve as surrogates for intrinsic noise strength.

**Section 7.1 Estimating** $\hat{ћ}$ **from Neural Recordings**

Our proposal to estimate $\hat{ћ}$ from patch-clamp membrane-potential variance together with an effective inertial parameter *m* follows the program introduced by Ghose and Pinotsis [2]. We adopt their rationale while adapting details to our dataset.

**Figure 1 caption, sentence added**

This pipeline follows the standard Nelson–Madelung construction; related expositions include Ghose and Pinotsis [2].

**Reference [2] was updated from the preprint to the final article**

Ghose, P.; Pinotsis, D.A. The FitzHugh–Nagumo Equations and Quantum Noise. *Comput. Struct. Biotechnol. J.* **2025**, *30*, 12–20. https://doi.org/10.1016/j.csbj.2025.02.023. 

The authors state that the scientific conclusions are unaffected. This correction was approved by the Academic Editor. The original publication has also been updated.
